# A qualitative exploration of Chinese rural older adults’ adaption experience to disability in Henan Province

**DOI:** 10.1186/s12889-023-15425-0

**Published:** 2023-03-17

**Authors:** Mengke Gao, Yan Zhang, Yutong Tian, Yue Gao, Xiaohua Li, Yixin Lu

**Affiliations:** School of Nursing and Health, Zhengzhou University, 101 Science Avenue, High-Tech Zone, Zhengzhou City, Henan Province China

**Keywords:** Rural areas, Disabled older adults, Adaptability, Qualitative research

## Abstract

**Background:**

The global population is ageing in a serious way and the number of disabled elderly people is increasing. Disability is a combination of physical and functional impairments, activity limitations, and social participation restrictions that significantly affect the quality of life of older adults. This study used the Roy adaptation model to examine the adaptive strategies of rural disabled elderly.

**Methods:**

An interview outline was prepared based on the Roy Adaptation Model, in-depth interviews were conducted with eligible rural elderly with disabilities using purposive sampling. Interview data were analyzed using the colaizzi method to obtain relevant themes and sub-themes of the adaptation experience.

**Results:**

Fifteen eligible disabled elderly participated in the interview, with an average age of 73.7 years old, showing different adaptation experiences in different aspects, a total of 5 themes and 18 sub-themes were extracted: (a)physiological function adaptation: learning to monitor physiological indicators, active medical compliance behavior, active rehabilitation exercise, adjusting lifestyle and coping with failure, (b) self-concept adaptation: adjustment of gratitude mentality, self-consolation, transferring the attention, seeking emotional comfort, and negative emotional response, (c) role function adaptation: positive self-care role, negative family role and escape of social role, (d) interdependence adaptation: actively seeking support and complex social coping, and (e) adaptation influencing factors: personal factors, caregiver factors and the policy factors.

**Conclusions:**

The disabled elderly show different adaptation strategies in four ways, and are affected by personal factors, caregiver factors and policy factors. A multi-faceted support system for the disabled elderly is recommended, and the caregivers should be trained in all-round care knowledge and skills.

## Introduction

The increase in the aging global population is the most challenging social problem in the world [1].The number of people aged 60 years or older will rise from 900 million to 2 billion between 2015 and 2050 (moving from 12 to 22% of the total global population) [2]. As life expectancy increases, the number of older people with disabilities at risk of chronic illness or injury also inevitably increases [3]. Disability poses an important challenge to countries all over the world since it affects more than 15% of the global population (2019) [4]. In 2020, a study conducted in eight low- and middle-income countries (China, Cuba, Dominican Republic, India, Mexico, Peru, Puerto Rico, and Venezuela) found that the proportion of remaining life spent disability-free at age 65 ranged from the lowest in Peru (76% for men and 69% for women) to the highest in China (92% for men and 89% for women) [5].At the end of the last century, China entered an aging society, with a rapidly growing, aging, disabled and empty-nest population. China has the largest population of older people with partial or total disabilities in the world [6]. In 2021, the results of the seventh national census showed that 18.70 percent of the population aged 60 and over in China, with over 200 million rural older adults [7].The serious population aging trend aggravates the physiological decline of the older adults, there are about 44 million disabled and semi-disabled elderly in China, and the total number of rural disabled people exceeds 8 million. It is expected that older disabled adults population in China will reach 77 million by 2030(2021) [8], with rural disabled people accounting for the largest proportion.

The Chinese government is very concerned about the disabled elderly, having proposed that improving and safeguarding their living conditions is an essential part of promoting the well-being of the people [9].In the Strategic Plan for Rural Revitalization (2018–2022), it is mentioned to support the construction of rural pension service systems for the disabled and semi-disabled elderly and to improve the level of mutual pension services [10]. Therefore, paying attention to the quality of life of rural disabled elderly is an important link to realize ‘healthy China’ and ‘ healthy countryside ‘. It will also be useful for research on disabled elderly in other countries/regions of the world.

The disability adaptation of the rural older adults needs to be paid attention to. Adaptability is a kind of ability that individuals adjust themselves to the new human environment, new communication groups, new behavior and target requirements [11]. According to the ICF (International Classification of Functioning, Disability and Health), an disabled person is one who, due to old age, illness or disability, has to be assisted by others in activities of daily living such as eating, bathing, dressing, toileting, continence control and indoor activities, or older people who are completely dependent on the assistance of others. Of the six activities listed above, one to two “can not do” is defined as “mild disability”, three to four “can not do” is defined as “moderate disability”, five to six “can not do” is defined as “severe disability” [12]. Disability is a combination of physical structural and functional impairment, activity limitation and social participation restriction in interaction with health conditions, resulting from the individual’s functional status, physical environment, cultural environment and policy environment [13]. Functional impairment is positively correlated with the increasing age of older people [14]. Therefore, the adaptability of disabled older people should be taken into account. Due to the loss of productivity resulting from partial or complete loss of self-care, coupled with low rural income levels, low medical resources and part of the traditional thinking of rural elderly people, rural disabled elderly people are unable to adapt to the physical changes caused by their disability and are more prone to physical, psychological, emotional and social maladjustment [15].Therefore, the adaptability of the elderly disabled in rural areas is an issue that needs urgent attention. However, research on the disabled elderly has mainly focused on care models, and studies on their adaptability have only been conducted on myocardial infarction patients and cancer patients in China [16, 17], with less research on the level of adaptability and factors affecting the disabled elderly in rural areas, and it is urgent to carry out relevant studies.

At present, much of the research on the rural older adults with disabilities has been based on the quantitative method [18]; however, a fixed questionnaire can often ignore the indelible individual experience of the interviewees. To obtain more vivid and richer knowledge, we adopt an inductive approach in this paper, using a qualitative method to search the subjective disability adaptation experiences of the disabled elderly in rural China through the collection of in-depth interviews. An in-depth interview is a type of unstructured, direct, deep, and one-to-one interview, which is an appropriate way to collect data on the potential motives, experiences, attitudes, and emotions of the respondents regarding a certain issue [19].

Based on the Roy Adaptation Model, this study interviewed the disabled elderly in a rural county in Henan Province to analyse this population’s experiences of adaptation to disability.

## Methods

### Study setting and participants

Guided by the qualitative research method, this study analyzed the adaptation experience of rural disabled elderly through in-depth interviews. And this study referred to the qualitative research report standard prepared by O’ Brien et al. [20] as the guidance for research design and development. The principles of informed consent, no harm, and participant confidentiality were strictly followed in our study during the in-depth interviews. The research protocol was approved by the school’s ethics committee. (ZZUIRB-2022–20).

We recruited participants through the local village committees, an administrative institution in rural China responsible for the provision of social welfare and social services for the rural disabled and rural older adults, including cash allowances, health rehabilitation,care services, and medical reimbursement support [21]. Henan Province is the most populous province in China and the third most populous province in terms of resident population, and the evolution of the stages of population ageing is in line with national trends. In 2020, the province’s population aged 60 and above reaches 17.964 million, accounting for 18.08% of the province’s resident population [22]. In recent years, the level of population ageing in rural Henan Province is higher than that in urban areas, and the number of disabled and semi-disabled elderly people is on the rise [23]. Therefore, we chose rural areas in Henan Province as our study site.From June2022 to July 2022, the researcher used a purpose sampling method to investigate the disabled elderly in a administrative village in a county of Henan Province(16.91% of older people in the area), where villagers are mainly engaged in farming and some young people work outside the home, representing most rural conditions in China [23].

The participants were recruited through purposive sampling, considering specific criteria related to the research objectives. The criteria were as follows:

- Over 60 years old;
- The degree of disability reaches at least “mild disability” (in accordance with ICF, eating, dressing, getting out of bed, toileting, indoor walking, bathing six indicators, one to two “can not do” is defined as “mild disability”);
- having certain language expression ability(ability to complete listening, speaking, reading and writing independently);
- No previous cognitive impairment or psychosis (one or more of the functions of memory, spatial ability, judgment and calculation are impaired);
- Voluntary participation in this study;
- Not in the acute phase of the disease(the elderly with unstable condition within two weeks of illness, such as fever, acute respiratory infection, asthma attack, etc.); or chronic infectious diseases (such as tuberculosis, hepatitis B that can spread between people).

The study began with an introduction to the purpose and methods of the study, an explanation of the study, and a promise to participants that all personal information and conversations would be confidential and secure, and that participants could withdraw from the study at any time without consequence. The sample size for this study was referenced to the principles of qualitative research and the inclusion of new subjects was stopped when the data analysis was saturated and no new themes emerged [24]. Ultimately, a total of 15 eligible rural elderly people with disabilities participated and completed the study, and all signed an informed consent form.

### Conceptual framework

**Fig. 1:**
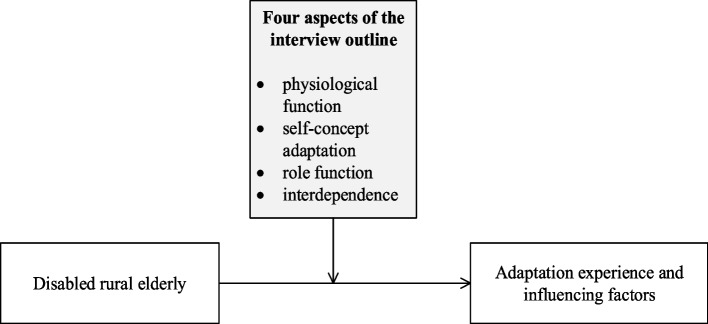
Conceptual framework

Roy’s adaptation model was proposed by the renowned theorist Roy in the 1960s [25], argues that stimulus input affects the cognitive/regulatory system of an individual and leads to the cognitive adaptation process. The perception of illness in the internal environment interacts with this process, leading to dynamic adjustments in the body’s physiological functioning, self-concept, role functioning and interdependence, with the latest red manifestations being behavioural adaptation or maladaptation. These four adaptation strategies suggest that an outline of an interview on the adaptation experiences of rural older people with disabilities can be developed from these four areas. The conceptual framework is shown in Fig. 1.

### Procedure

All researchers were trained in qualitative methods and interview techniques. Firstly, the researchers developed an interview guide based on Roy’s adaptation model [25], combined with expert opinion—focusing on asking the elderly about their reactions and coping styles before and after disability—and used language that was easily understood by the rural elderly, focusing on four aspects of the adaptation experiences of the disabled elderly. Through interviews with two cases of rural disabled older people, we found that different older people had different experiences of adaptation influenced by different factors. Based on this insight, the interview outline was adapted after discussion and influencing factors were added after each question. The final version of the interview guide is shown in Table 1. Demographic information was collected at the beginning of the formal interview, including information on gender, age, marital status, and educational attainment.

**Table 1:** Interview guideline

Final edition
**Question1:**What difficulties have been encountered in your life after illness? How do you deal with these difficulties? What factors affect you to deal with these difficulties?
**Question2**: How your mood changes since the illness? How do you adjust your mood? What factors affect your emotion regulation?
**Question3**: What are your changes in self-care roles, family roles, and social roles after your illness? How do you deal with the role changes before and after illness? What factors affect your response to role change?
**Question4**:What social support do you perceive after illness? What changes have taken place in interpersonal relationship? How do you deal with these changes? What factors affect your response to these changes?

### Data collection

The interviews were conducted using a face-to-face semi-structured interview method. Prior to the interviews, the researcher made appointments by phone and text messages. The location and time of the interviews were chosen to suit the convenience of the interviewees. The interviews were all conducted in the patient’s home to ensure a relaxed and comfortable environment, with no other people present at the interview.The entire interviews wss recorded with the consent of the interviewee; the interview was asked in an open-ended questioning style, allowing the interviewee to freely express his or her true experiences and feelings. The interview questions and the interviewer’s attitude were neutral to avoid bias against the participants. During the interviews, the researcher carefully observed and recorded the subtle facial expressions and movements of the interviewees. The average interview time was 35 min.

### Data analysis

After each interview, the researcher transcribed the interview transcripts verbatim within 24 h, collated and documented each interviewee’s interview transcripts, and conducted data analysis and collection. The interview data were analysed using the Colaizzi seven-step analysis method [26]: a) detailed transcription and careful reading of all interview materials; b) selection of summaries and meaningful expressions that corresponded to the research phenomenon; c) distillation of meaning from the meaningful expressions; d) search for common conceptual or meaningful features to form themes, thematic groups and categories; e) linking themes to the research phenomenon to a complete narrative; f) stating the essential structure of the phenomenon; and g) returning the results to the interviewee to verify the authenticity of the content.

## Results

### Participants

The characteristics of the 15 participants are presented in Table 2. The adaptation experience of rural disabled elderly is finally summarized as the following 5 themes and 18 sub-themes in Table 3.

**Table 2:** Characteristics of the participants

Characteristics	Number of participants (%)	Characteristics	Number of participants (%)
Age groups		Education level	
60–69	4(26.67)	Non-formal education	2(13.33)
70–79	8(53.33)	Elementary education	4(26.67)
80–89	2(13.33)	Middle school education	7(46.67)
90–100	1(6.67)	High school education	2(13.33)
Gender		Marital status	
Male	8(53.33)	Married	8(53.33)
Female	7 (46.67)	Widowed	7(46.67)
Degree of disability		Length of Disability/years	
Mild Disability	8(53.33)	0–5	8(53.33)
Moderate Disability	4(26.67)	6–10	4(26.67)
Severe Disability	3(20.00)	11–15	3(20.00)

In accordance with ICF, eating, dressing, getting out of bed, toileting, indoor walking, bathing six indicators, one to two “can not do” is defined as “mild disability” ‘, three to four “can not do” is defined as “moderate disability”, five to six “can not do” is defined as “severe disability”

**Table 3:** Rural disabled elderly adaptation experience theme

Theme	Sub-theme
Physiological function adaptation	a) learning to monitor physiological indicators b) active medical compliance behavior c) active rehabilitation exercise d) adjusting lifestyle e) coping with failure
Self-concept adaptation	a) adjustment of gratitude mentality b) self-consolation c) transferring the attention d) seeking emotional comfort e) negative emotional response
Role function adaptation	a) positive self-care role b) negative family role c) escape of social role
Interdependence adaptation	a) actively seeking support b) complex social coping
adaptation influencing factors	a) personal factors b) caregiver factors c) policy factors

Five themes were generated in the interview. The first four themes were the adaptive experiences of disabled elderly people in physiological function, self-concept, role function and interdependent mode, and the sub-themes were specific behaviors of adaptive mode or maladaptive outcomes. The fifth theme is the reasons that influence disabled elderly people to have these different adaptive experiences. The three sub-themes are explained in detail from personal reasons, caregiver reasons and policy reasons.

### Theme1: Physiological function adaptation

Physiological function adaptation refers to the ways in which rural disabled older adults cope with changes in their physical functioning, including physiological index response and physiological symptom response. Five sub-themes emerged in this theme.

#### Learning to monitor physiological indicators

Seven rural disabled elderly mentioned that they learned to measure their blood pressure, blood sugar and other indicators after illness, and tried to maintain these physiological indicators related to disability at normal levels.They were mostly mildly disabled elderly, or highly educated severely disabled elderly.

“I have an annual medical check-up and usually take my own blood pressure and blood sugar at home. If there are any physical symptoms, a call to the doctor can help.” (female,85 years old, widowed, severe disability, high school education)

#### Active medical compliance behavior

Ten respondents with disabilities due to chronic illnesses reported that they always followed medical advice, adhered to the principles of medication and coped with changes in physical function through specialist treatment.Most of these people were widowed, and the degree of disability was mild or moderate.

“I take my medication regularly as required by my doctor, and because of my high blood pressure and thick blood lipids, I have to take several kinds of medication, all of which I take as required by my doctor. I had a stroke mainly because I didn’t pay attention to my high blood pressure before, so I am now very careful to take my blood pressure medication as required.” (female,75 years old, married, mild disability, non-formal education)

#### Active rehabilitation exercise

Seven out of 15 disabled elderly in rural areas reported that physical changes such as reduced mobility and swallowing disorders that affect self-care can occur after illness. They would choose to actively exercise their physical activity and swallowing ability, reflecting the rural elderly’s quest for a healthy physique. These people were mostly men, as well as married elderly people with mild disability, or highly educated elderly people with severe disability.

“Doctors said that my swallowing function is affected. I often practice at home, repeatedly do pharyngeal movements, do tongue activities... And I can’t move this hand, now better, because I grabbed my own hands every day bed exercise, clench fists twenty times, pull arms twenty times, and then lying in bed, legs up in the air to learn to walk fifty times (while demonstration )...” (male,87 years old, widowed, severe disability, high school education)

#### Adjusting lifestyle

Eight out of 15 respondents reported changing their bad habits after becoming ill. The quality of sleep decreased for some people. Some older adults with moderate to severe disabilities have severe activity limitations, resulting in increased bedtime and slower bowel movements, often leading to constipation. Some elderly families with disabilities cautiously adjusted their dietary strategies. These people showed different degrees of disability, with mild disability and male majority.

“After getting sick, I quit smoking and drinking, and I don’t stay up late. ” (male, 61 years old, married, mild disability, elementary education)

“I usually don’t eat vegetables. For a period of time, I always constipation. My daughter always makes vegetables in different ways, squeezes them into juice and makes them into soup... and tries to make me eat more vegetables and regulate them.” (male,87 years old, widowed, severe disability, high school education)

#### Coping with failure

Ten out of 15 disabled elderly said they feel difficult to adapt to the decline of thinking ability, unable to cope with and choose to compromise. Among these people, 3out of 4 are women with low educational level.

“I used to play chess with my friends, since the surgery, the brain can’t think about things, a thought is blind, I don’t want to think about anything...” (female, 70 years old, widowed, mild disability, elementary education)

### Theme 2: Self-concept adaptation

Self-concept is people’s emotion, confidence and evaluation of themselves at a certain time. Self-concept adaptation reflects the individual’s response to psychological and mental health changes. This theme includes five sub-themes.

#### Adjustment of gratitude mentality

We found that 9 respondents can adjust their negative emotions with a heart of satisfaction and gratitude. 6 respondents felt happy and moved because they felt the care of their relatives in life after disability. These people were mostly married elderly people, or widowed elderly people with higher education levels.

“I felt that my daughter-in-law was very kind to me, and I was moved... I can’t walk, my grandson hugged me downstairs. Without elevators, he held me in his hands and was tired to breathe. I said, having a strong man, I was so moved.” (female,85 years old, widowed, severe disability, high school education)

#### Self-consolation

The interview found that 8 rural disabled elderly often persuade themself to keep positive after illness, maintain an optimistic attitude, make their usual treatment of disease.Most of these people were married, and had a shorter duration of disability.

“I sometimes think that it won’t be worse, so just rest and recuperate. The kids grow up and don’t need me to care. Anyway, life is much better than when I was a kid.” (male,72 years old, married, moderate disability, middle school education)

#### Transferring the attention

Seven disabled elderly in rural areas showed that they can relieve negative emotions by developing new interests and learning new things to transfer their attention. These people were mostly mildly disabled elderly.

“It’s a little hard to talk after a stroke. And my hearing is poor. It’s very difficult to communicate with others. So I usually spending time painting alone. I paint on the blackboard with chalk, which make me feel happy..” (female,75 years old, widowed, mild disability, elementary education)

#### Seeking emotional comfort

The disabled elderly will have feelings of loneliness, inferiority and self-accusation. They tell their friends about their feelings to alleviate their emotions. These people were widowed and disabled for a long time.

“I have a friend, who often came home to chat with me, I often tell her my feelings which I don’t want to say to the family members, after that she will comfort and understand me, let me feel not lonely.” (male, 92 years old, widowed, moderate disability, middle school education)

#### Negative emotional response

Eleven out of 15 respondents had long been in negative emotions since their disability, including guilt and self-accusation, pessimistic depression and so on. And they cannot release these emotions successfully. These people were widowed elderly, mostly moderate disability and severe disability.

Ten rural disabled elderly felt remorseful for their family economic burden due to long illness.

“I feel like I’m a burden in my family. Every day I exist adds a lot of burden to children.” (male, 87 years old, widowed, severe disability, high school education)

Seven respondents were disappointed and depressed because of limited activity or prolonged disease.

“I lie in bed day by day because I can’t move, and I can’t sleep night by night. It’s better to die to think about what...” (male, 87 years old, widowed, severe disability, high school education)

### Theme 3: Role function adaptation

Role function adaptation includes coping with self-care role, family role and social role change of disabled elderly. This theme contains three sub-themes.

#### Positive self-care role

Rural older people have an increased ability to care for themselves after illness. Most older people with disabilities love life and show positive self-care skills. This is demonstrated by paying close attention to their own health, actively cooperating with treatment, seeking help in a timely manner, learning about relevant health issues in a variety of ways, and developing self-care skills. These people were mostly married, mildly disabled elderly, or widowed elderly with junior school or high school education.

“I often remind myself as a patient, the heart is not good, so don’t angry with others”. (female,76 years old, widowed, mild disability, middle school education)

“I usually pay attention to health knowledge on TV, and the blood glucose was well controlled because I don’t eat sweet food.” (female, 85 years old, widowed, severe disability, high school education)

“I especially care about my body, a little uncomfortable I will ask the people around me for help.” (male, 61 years old, married, mild disability, elementary education)

#### Negative family role coping

Eight older adults were reluctant to participate in the management and decision-making of family affairs after disability, and their concern for family members was also reduced. Only 4 out of 15 older adults were willing to do some housework that they can.

Seven out of 15 older adults lost their ability to work due to limited activities. They needed to be care all time at home and completely lost the ability to help families share responsibilities.

“I’m in a bad mood after I get sick. I have no mood to do anything for my family.” (female, 61 years old, married, mild disability, elementary education)

There were also 8 out of 15 respondents who trid their own affairs within the scope of ability after disability, reflecting their family participation.

“I can’ t do most of the housework after I get sick. I can only do some small things, such as sweeping the floor.” (male, 60 years old, widowed, mild disability, elementary education)

#### Escape of social roles

Rural disabled elderly rarely participate in social activities due to cultural constraints and physical reasons after illness. 5 disabled elderly indicated that they were not concerned about national affairs, and they had no TV or radio at home, unwilling to know the latest pension policy and unwilling to participate in collective activities. These elderly people were primary school educated or uneducated.

“I only watch TV drama, not want to watch news at all,and I am just a sick rural old man, I do not care about national affairs, just want to have a good life.” (male, 78 years old, married, mild disability, elementary education)

“I don’t have much experience sharing with young people either. Now young people have their own opinions.” (female, 61 years old, married, mild disability, elementary education)

### Theme 4: Interdependence adaptation

Interdependence refers to individuals develop relationships with others to meet emotional, developmental and resource needs. Interdependence adaptation refers to the situation of rural older adults who obtain social support and maintain interpersonal relationships after disability. There are two sub-themes in this theme.

#### Actively seeking support

Actively seeking support is manifested in many aspects: seeking family support, seeking relatives and friends support, seeking village committee support, seeking village doctor support, perceived policy support. Those with these presentations tended to have mild or moderate disabilities, and there were more widowed older people than married older people, showing different levels of education.

“After being ill, I could not engage in physical labour. Relatives came to help us harvest corn and grow wheat, thanks to the help of relatives, or the crops were wasted.” (male, 92 years old, widowed, moderate disability, middle school education)

“Our neighbor has a van, and he drives us to the hospital for several times, which is so convenient.” (female, 77 years old, widowed, mild disability, elementary education)

“The first thing I thought of when I was sick was going to our village clinic, just take some medicine, I will be fine. Sometimes I can’t go, but I call the village doctor, he rides a bike with a medical bag and comes to my house.” (female, 76 years old, widowed, mild disability, middle school education)

“Whenever I have trouble at home I call the village cadres. Everyone is very good to me, take care of me as family members.” (male, 61years old, married, mild disability, high school education)

#### Complex social response

Complex social response is manifested as social interaction with peers, communication with relatives and friends, and social interaction with strange sick friends.

“I always feel uncomfortable communicating with my peers since I was sick, I wouldn’t like to talk with them, sometimes I envy their physical condition.” (male, 78years old, married, mild disability, elementary education)

“There are more contacts with relatives after illness, because I often need their care. At the beginning, everyone was very positive, they knew I was sick and often came to see me, asking me if I had enough money, but I was too embarrassed to bother them, thinking that everyone was too busy to visit me every day.” (female, 77years old, widowed, mild disability, elementary education)

### Theme 5: Factors influencing for adaptation

This theme includes three sub-themes.

#### Personal factors

Personal factors include disability duration, psychological resilience, self-efficacy and family income.

In the interview, 5 out of 15 disabled elderly said that the longer their illness, the more able to adapt to the life after illness.

“When I have just checked out this disease, I am very irritable, often angry, and become very irritable, and I cannot control myself, but now I have accepted the fact and am very peaceful to the disease.” (male, 65years old, married, moderate disability, elementary education)

Psychological resilience is an individual’s psychological capacity to cope with difficulties or stress. Rural elderly people with disabilities who adapt to different experiences present different psychological resilience.

“I adapt to this disease so well due to my personality. I used to be the village head, handled a lot of tricky things, experienced a variety of problems in the village, developed the strong psychological endurance.” (male, 72years old, married, moderate disability, middle school education)

We found that 6 out of 15 older adults showed positive self-efficacy after disability, whom were willing to actively share their own experience and actively participate in social interaction.

“I was a soldier, then a worker, and now I’m a civilian, still a disabled old man, but it doesn’t matter, my friends and I talk a lot about what happened in the past, about society now, about what I’ve learned about my life, and about this illness.” (male, 61years old, married, mild disability, high school education)

We found that rural disabled elderly with high family income showed good adaptability.

“My husband was a primary school teacher when she was younger and now receives a monthly pension. Sometimes the children give some change and we receive a pension every month. I bought my electric wheelchair with the money we both saved.” (female,70years old, widowed, mild disability, elementary education)

#### Caregiver factors

The caregiver factor is mainly whether the caregiver has received training.The trained caregivers make the disabled elderly show good adaptability.

“My daughter-in-law has ever trained in the town especially for caring me, and she have been taking care of me since I am sick, feeding, scrubbing, taking medicine, also teaching me some rehabilitation exercises. With her careful care, I recovered quickly.” (male, 65years old, married, moderate disability, elementary education)

#### The policy factors

The policy factors include social security policy and medical insurance policy. The Chinese government attaches great importance to social security for the vulnerable elderly, which facilitates the adaptation of the disabled elderly in rural areas.

“The national policy is very good, especially for us elderly people, we get a pension when we are over 60 years old, those in our village who are over 60 years old receive more than 100 yuan per month and those over 70 years old receive 200 yaun per month, this policy is very good and protects the livelihood of our elderly people.” (female, 75years old, married, mild disability, non-formal education)

“I was afraid of getting sick and spending money so I didn’t want to go to the hospital, but we have the New Agricultural Cooperative Medical Insurance and we can get 80% reimbursement for hospitalisation at the county hospital, which saves us a lot of money and makes me feel a lot more relieved.” (male, 75years old, widowed, severe disability, elementary education)

## Discussion

This study explored the adaptation experience of rural disabled elderly. The disabled elderly in rural areas showed different experiences in different aspects of adaptation.

### Different adaptation strategies

In the physiological functioning adaptation experience, our study mainly found that rural disabled elderly took various measures to cope with physiological changes, including monitoring of physiological indicators, active compliance behaviours, active rehabilitation exercises and lifestyle adjustments, which is consistent with the findings of a similar study by Bockwoldt. Bockwoldt et al. [27] described adaptation strategies for elderly with diabetes in terms of physiological functioning by incorporating diabetes-related activities into existing lifestyles, such as monitoring blood glucose, observing symptoms, and taking and/or adjusting oral or injectable medications; studies by Li H [28] also found that rural elderly with disability experience self-lifestyle adjustments, positive health beliefs, and increased self-management of health conditions. This suggests that rural elderly people with disabilities are actively adapting themselves to cope with physical changes, in line with the concept of "active ageing" advocated by the Chinese government [29]. From this survey, we learned that the elderly in rural areas have purchased blood pressure monitors to measure their own blood pressure, reflecting the strong health awareness of the elderly in rural areas, but this is inseparable from the country’s strategic plan for economic development, achieving comprehensive poverty eradication and improving the living standards of the grassroots [30]. Our secondary finding is some people said they are unable to cope with physiological changes, whom were mostly women or elderly people with low levels of education, suggesting that gender and education are relevant to the physical adaptability of the disabled elderly. The main findings and secondary findings are complementary to each other, which together constitute the diversity of physiological function adaptation and the different outcomes of adaptive behavior and ineffective adaptation, which is consistent with the two adaptation results in Roy ‘s adaptation model. Caregivers should pay more attention to these people with ineffective adaptation, analyze the reasons for their inability to cope, and help them create adaptive environments and opportunities.

In self-concept adaptation, our interviews mainly found that in the face of psycho-emotional changes, rural disabled elderly coped through self-adjustment, distraction and seeking help from others. This is consistent with the findings of He et al. [31], which showed positive self-efficacy and stress coping abilities of some of the disabled elderly. Our secondary finding was that other disabled older people were unable to demonstrate good self-adaptation, to mitigate the psycho-emotional changes associated with their disability, and to get along well with their surroundings. The majority of this group are widowed or severely disabled, showing that marital status and degree of disability are related to the self-concept of adaptation of the disabled elderly. Some studies have pointed out that when negative emotions such as guilt and low self-esteem, depression and depression are not resolved, as well as morbid shame such as remorse and guilt, fear and despair cannot be relieved, and stress is not managed properly, rural elderly people may engage in suicidal behaviour [32], which may be related to their lack of mental health knowledge and less attention to this issue of their own mental health. It is suggested that the government should pay attention to the mental health problems of rural disabled elderly people, especially for the elderly who are widowed or severely disabled, strengthen the training of medical personnel in rural health centres and village clinics on mental health knowledge, as well as increase the popularisation of mental health knowledge among rural residents, set up a psychological counselling room in each village, etc., to pay attention to the mental health status of each disabled person attending the clinic and increase their adaptability to mental health.

In the role-functional adaptation experience, our study mainly found that rural disabled elderly showed positive self-care roles and active adjustment of themselves to disability in terms of health management, which is consistent with Bo [33].This may be due to the improvement of living standards so they can pay more attention to their own health than before, also reflects the desire for health of the disabled elderly in rural areas. In addition, our study also found that regarding family roles and social roles, the disabled elderly showed negative avoidance, which is consistent with the results of similar studies [34], which may because the limitation of resources in rural areas and the age of the older adults make them at the edge of society and are unwilling to participate in social things. The education level of this segment of the population is usually low, limiting their ability to learn new things. Guan’s research showed that with the progress of disease, patients will be aware of their responsibilities and obligations to their families so as to actively face life and self-growth, which is related to their self-efficacy [35].Patients will actively self-efficacy can due their responsibilities and obligations to the family, suggesting that caregivers should pay attention to the psychological characteristics of the disabled elderly, mobilize their enthusiasm for life, encourage them to realize their self-efficacy and actively participate in family affairs and social activities.

In the interdependent adaptation experience, our study mainly found that most rural disabled elderly received care and financial support from family, relatives and friends, and society, which is consistent with the study of Huo’s [36]. Social support plays an important role in the health promotion of patients, which can reduce the impact of various negative stresses, alleviate physical and psychological barriers, and enhance patients’ social adaptation [37, 38]. We also found that there are different situations when the rural older adults dealing with the relationship with different people such as friends, family members, peers and village doctors, and their social adaptation is terrible. Some studies showed the same view [39],some disabled elderly reduce social activities or even social willingness due to decreased activity ability or psychological reasons. This may also be caused by the physiological and psychological changes caused by disability. The disabled elderly are unable to participate in social activities due to malnutrition, inability to sit long, need to be accompanied outside, visual and hearing loss, urinary incontinence and other reasons, resulting in a decline in enthusiasm for participation, thereby reducing the demand for social participation [40].Leisure activities were important for the promotion of physical and mental health [41, 42],it is necessary to emphasize the importance of socialization with family and friends and leisure activities in assisting the older adults in rural areas with disability issues.Leisure activities for older adults rural people with mobility difficulties and living in areas with limited infrastructure development could be directed towards mental activities [43]. In addition, studies have shown that peer support can improve the self-management level of the older adults [44]. Similar cultural backgrounds and rehabilitation experiences enable rural disabled elderly neighbours to understand each other’s inner feelings and real needs [45], which is the principle of the ‘peer effect’ [46, 47]. This suggests that patients from neighbouring villages with the same illness can share their experiences, do rehabilitation exercises together, monitor each other, and improve the health management of rural disabled older people, as well as help them to establish new social circles, participate in new social activities, regain social confidence and improve their social adaptability.

The factors that effect these adaptation strategies.

People are in a state of constant response to environmental changes, and the result of interaction and integration between people and the environment is adaptation. The adaptation process of the disabled elderly is affected by some environmental factors such as personal factors, caregiver factors or policy factors.When analyzing the factors that affect the adaptation strategies of disabled elderly, we mainly found that disability duration, family income, psychological resilience and self-efficacy were the personal factors affecting the adaptation experience of rural disabled elderly.

The interviews revealed that some of the disabled older people had become more comfortable with their disabilities as they grew older, possibly because they had been exposed to more knowledge about illness and coping skills as they grew older, and were more comfortable with their surroundings. The elderly with higher family incomes were more able to afford health products, especially assistive living devices such as electric wheelchairs, which can help them get along better with their surroundings. Psychological resilience refers to an individual’s ability to adapt positively and cope effectively in the face of adverse circumstances such as stress, pressure and adversity, and is conducive to an individual’s health and positive adaptation [48]. Disabled elderly people with different psychological resilience in this study showed different adaptive abilities. Song [49] showed that disabled elderly people have difficulty coping effectively with various stresses and pressures due to their high level of physical illness and low ability to perform daily activities, as evidenced by a lack of confidence and patience, poor mental capacity and increased negative emotions and maladjustment. This shows that psychological resilience is crucial to the level of adaptation of elderly people with disabilities, suggesting that attention should be paid to the psychological resilience of elderly people with disabilities in rural areas and timely psychological care guidance should be provided to caregivers. Self-efficacy refers to the intensity or degree of individual confidence in their ability to complete tasks and achieve goals [50, 51], which has a positive predictive effect on interpersonal adaptability, role adaptability and life self-care adaptability [52]. It is suggested that caregivers are prompted to help disabled older people to identify their self-worth, assess their abilities, give full play to their self-efficacy and facilitate their role adaptation, based on their own occupations and personality traits when they were younger.

In addition, our study also found that gender, education level, disability level and marital status can also affect the adaptation. In physiological function adaptation, we found that male disabled elderly were more willing to take active rehabilitation exercise and were willing to adjust their lifestyles to cope with the physiological changes caused by disability, while women are more likely to cope with failure.Studies have shown that older rural women are less physically fit, at higher risk of disability and relatively less resilient to disability due to a variety of physical or psychological factors [53]. In our study, the mildly disabled elderly showed positive adaptive responses in four adaptation methods, such as more positive compliance behavior, distraction, positive self-care behavior, and active pursuit of social support, which may be due to the fact that the self-care ability of the mildly disabled elderly is not completely lost and there are significant differences in physiological function, vitality and emotional function among the older adults with different levels of disability. As the degree of disability increases, the physiological adaptations of older people decline [54]. In addition, high levels of education had a positive impact on the adjustment of disabled older people in our study. Some older people with moderate disabilities who had a high level of education performed better in monitoring physical indicators, gratitude adjustment and positive self-care. It may be because the older adults with high educational level are good at self-conform, have correct disease cognition, and show high self-efficacy, so they can better adapt to the changes caused by disability. In our study, the elderly with spouses performed better in positive compliance behavior and self-consolation, while the widowed elderly performed worse in self-concept adaptation.

Caregiver factors were the knowledge and skills of the caregiver and whether or not they have received training. This interview revealed that the influence of different caregivers on the disabled elderly is completely different. The trained caregivers of the disabled elderly pay close attention to their health and mental conditions, and make reasonable arrangements for their outings, nutritional intake, adequate companionship and psychological support, therefore, these disabled elderly often feel happy and moved. This suggested that the caregivers play an important role in the psychological adaptation process of the disabled elderly, which is consistent with Liu’s [55]. Rural disabled elderly depend on caregivers for long-term care because of their poor self-care ability; meantime, they spend most of time at home due to limited mobility, and loneliness caused by long-term lack of peer communication has resulted in strong psychological dependence on caregivers [36]. Therefore, caregivers are crucial for the physical and mental health development of rural disabled elderly. The study pointed out that the family caregivers of the disabled elderly have become the mainstay of the long-term care system [55], and played the roles of daily caregivers, collaborative treatment, safety defenders and psychological comforters of the disabled elderly. However, the care needs of rural elderly with disabilities are special, long-term and complex, while caregivers are mostly non-professionals such as spouses and children, and are old and of low professional level. Therefore, their ability to receive new knowledge is limited, and their caregiving ability cannot meet the care needs. Mao pointed out that comfortable nursing training for caregivers of disabled elderly can improve their care skills and self-confidence, and also improve the quality of life of disabled elderly [48]. It is suggested that the caregivers should be trained in comprehensive care knowledge and skills, such as blood pressure monitoring, feeding skills, medication precautions, rehabilitation training skills, skin care, oral care, and psychological care. It can also empower the caregivers to have more knowledge and ability to be competent for the role of caregivers, so as to improve the adaptability of the disabled elderly.

Policy support was an important factor for the adaptation of rural disabled elderly, and other scholars also proves this view. Szanton noted that the elderly who receive social security and services have a higher level of disability adaptation, which is reflected in both psychological adaptation and disease adaptation [56]. Rural elderly with disability have a certain degree of financial burden due to the long duration of chronic diseases. Government-provided health insurance and Medicaid policies will reduce their disease burden and reduce their negative emotions such as guilt and self-blame due to the financial burden [57]. Choi suggested disability welfare programs should be provided to older adults who present with frailty [58]. It can be seen that the social security and services provided by the government play a positive role in supporting the improvement of the disability adaptability of the rural older adults.

### Suggestions on improving the adaptability of disabled elderly

Here are some suggestions to improve the adaptability of disabled elderly. At the national level, a multi-faceted social support system for the disabled elderly should be established on the existing basis. Firstly, laws and regulations relating to the disabled elderly should be improved, including regulations on long-term care insurance and discrimination against the elderly with disabilities; secondly, a mechanism should be established to promote rehabilitation activities for the elderly with multiple support from families, doctors and healthy peers, and an artificial intelligence-based “integrated network information security platform” should be improved for both urban and rural areas. Thirdly, the training of carers in all aspects of their skills should be strengthened. At the family and community levels, caregivers of the disabled elderly and rural grassroots health workers should pay more attention to women, the elderly with low education levels and the elderly with severe disabilities, using easy-to-understand language to popularise their health knowledge, help them build up a correct understanding of the disease, and enhance their confidence and courage to adapt. At the same time, we should pay attention to their psychological and emotional changes, help them better regulate their negative emotions in the process of adaptation and strengthen their courage to cope with difficulties, so that they can adapt better.

### Limitations

Firstly, the sample source has regional limitations. This study only interviewed rural disabled elderly in one city in Henan Province, and more research is needed on rural disabled elderly in other cities in Henan Province; secondly, qualitative research cannot analyse all the influencing factors. The analysis of factors influencing the adaptation of rural elderly with disabilities should be combined with quantitative surveys. Currently, there is no quantitative research on the adaptation of rural older people with disabilities and there is a lack of relevant assessment tools. As a next step, we plan to develop an adaptive capacity questionnaire for rural disabled older people in conjunction with Roy’s adaptive model as an assessment tool for quantitative research. Future research could incorporate progressive quantitative research to collect data and analyse influencing factors. Thirdly, this interview only interviewed older people with disabilities who have some verbal skills. Those with limited expressive language skills may have different experiences. The next step could be to explore the adaptation experiences and influencing factors of these individuals by interviewing their carers.

## Conclusion

The results of the interviews revealed that rural disabled elderly people present a multifaceted adaptation experience. In terms of physiological functional adaptation, medication and non-pharmacological treatments were chosen to adapt to physiological changes. About self-concept adaptation, negative emotions were alleviated through self-persuasion, distraction and help-seeking, but inability to cope with physical and psychological changes also occurred. For role function adaptation, positive self-care roles, negative family roles and avoidance of social roles are present. For interdependency adaptation, rural disabled elderly show active seeking of social support and complex interpersonal changes. Personal self-efficacy and psychological resilience, as well as the caregiving skills of caregivers, are important factors influencing the adjustment of older people with disabilities. It is recommended that a multi-dimensional social support system be established for the rural elderly with disabilities, and that training of carers in relevant caregiving knowledge and skills, with particular attention to the development of psychological caregiving skills, be strengthened, while promoting the reasonable development of the elderly with disabilities’ own self-efficacy.

## Data Availability

The qualitative datasets generated and/or analysed during the current study are not publicly available due to the data containing information that could compromise research participant privacy/consent, but are available from the researcher Mengke Gao (619418631@qq.com) on reasonable request, and subject to approval from the research committee of the Zhenghou University.
